# The Bioaccumulation and Health Risk Assessment of Metals among Two Most Consumed Species of Angling Fish (*Cyprinus carpio* and *Pseudohemiculter dispar*) in Liuzhou (China): Winter Should Be Treated as a Suitable Season for Fish Angling

**DOI:** 10.3390/ijerph19031519

**Published:** 2022-01-28

**Authors:** Yupei Hao, Xiongyi Miao, Mian Song, Hucai Zhang

**Affiliations:** 1 Institute for Ecological Research and Pollution Control of Plateau Lakes, School of Ecology and Environmental Science, Yunnan University, Kunming 650500, China; yphao66@126.com; 2 Key Laboratory of Karst Dynamics, MNR&GZAR, Institute of Krast Geology, Chinese Academy of Geological Sciences (CAGS), Guilin 541004, China; 3 Henan Xinweijie Technology Co., Ltd., Zhengzhou 450000, China; 4 Center for Hydrogeology and Environmental Geology, CGS, Baoding 071051, China; miansong@126.com

**Keywords:** wild fish, metals, seasonal changes, health risks, Liuzhou city

## Abstract

Wild fish caught by anglers were validated to be commonly polluted by metals, but their contamination status could be varied with changing seasons. To determine the seasonal variation in metal pollution and health risks in these fish, this study took Liuzhou City as an example to investigate the concentrations of eight metals in two dominant angling fishes (*Cyprinus carpio* and *Pseudohemiculter dispar*) collected, respectively, in winter and summer. The obtained results suggested the mean concentrations of metals in fish are overall lower in winter. Only Cr, Zn, and Cd in some fish were beyond the thresholds in summer. The significant correlations between fish length and weight and most metals suggested the biological dilution effect could exert its influence in winter. The similar distribution of metals in winter suggested that metal bioaccumulation should be manipulated by living habitats, while the inconsistent distribution of metals in summer may be related to the variation in feeding behavior. The metal pollution index (Pi) values were all below 0.2 in winter, which suggested no metal contamination in fish, but most fish were found to be mostly contaminated by Cr and Cd in summer, which was confirmed by their Pi > 0.2. The fish could be consumed freely in winter due to the total target hazard quotient (TTHQ) below 1, while the consumption of fish was not entirely safe in summer, particularly for children, due to TTHQ values that were generally beyond 1. Given the higher weekly recommended consumption of fish in winter, winter should be treated as a suitable season for fish angling.

## 1. Introduction

Fish are rich in protein, unsaturated fatty acids (DHA and taurine), and trace elements [1,2], the digestibility of which were confirmed to be significantly higher than that of beef, lamb, pork, and chicken [1,2], so that fish were commonly treated as the most vital source of various nutrients [3,4]. Previous studies reported that the regular consumption of fish cannot only prevent the development of heart and circulatory diseases, but also decrease the risk of stroke, coronary heart disease, diabetes, and hypertension [5,6]. Importantly, it can promote the normal development of the fetal brain, central nervous and visual systems [7]. Therefore, fish were considered to be popular aquatic products for human consumption [1,2]. However, with the widespread metal contamination in aquatic environments, one or several metals in fish were reported frequently beyond the safety thresholds across the world, particularly wild fish [2,3,8,9,10]. The consumption of contaminated fish would not only offset the benefits of fish consumption, but has a negative impact on human health [1]. Given the severe contamination in aquatic environments, wild fish are becoming a critical source of metals for human. Therefore, it is of great value to strengthen the investigation of metal pollution among wild fish.

In the context of the depletion of fishery resources and the sharp decline in biodiversity, fishing moratoriums and bans have become universal practices for the restoration of aquatic environments [2,11,12]. However, recreational fishing was rarely incorporated into the category of fishing bans, so that recreational fishing turned into the primary approach to obtain wild fish. Previous studies revealed that there are more than 220 million to 700 million active anglers all over the world [13], while more than 90 million fishing enthusiasts are active in China [1]. Although recreation is the primary purpose of fish angling, given the scarcity of wild fish, most anglers still consume the fish they catch or sell these fish to others [2,14,15]. However, the fish caught by anglers commonly have behaviors that they not only prefer to live around the river bank, but they prey on the aquatic biota gathering around the waterways adjacent to the sewage outlets [2], making these fish more susceptible to the contaminants emitted from the river bank and then subsequently accumulate metals [16]. Although the bioaccumulation of metals among fish could be mitigated by their species and size (length and weight) [1], the bioaccumulation of metals among these angling fish should be more likely dominated by their living and feeding behaviors around sewage outlets. Normally, the living and feeding behaviors of fish would be heavily regulated by climate change, which could be treated as a self-protection mechanism of fish to adapt to climate change [17,18,19]. The seasonal fluctuations in metal bioaccumulation among fish were reported to be significant and mainly related to their seasonal changes in living and feeding behaviors [17,19,20,21,22]. Although the frequent exposure of metal contaminants from sewage may alleviate the fluctuations in metal bioaccumulation among fish caught by anglers, the seasonal variations in living and feeding behaviors among these angling fish would be never absent, which inevitably exerts an impact on metal contamination among these fish. In order to guide the consumption of the fish caught by anglers, it is necessary to investigate the seasonal variations in metal contamination in these angling fish.

Liuzhou city is a typical industrial city in China and a regional economic center in Guangxi Province. However, the intense industrial operations have led to the very large emissions of effluents. Previous studies reported that the annual emissions of effluenst were considered to be more than 350 million tons, most of which should be blamed on the industrial operation of metal smelting and chemical, food, and paper industries [2]. Lastly, the discharged sewage converges into the most important surface runoff of Liuzhou City, causing metals to become the major contaminant types in Liujiang River [23]. However, the pollution of metals in these rivers has not deterred anglers from fishing. Many residents are still angling fish from this river. Given the prevalence of fishing, the consumption of these angling fish needs to be scientifically quantified. Despite the contamination of metals in these angling fish that have already been confirmed in our previous study [1,2], their safety for consumption has not been completely denied. The consumption of these fish within recommended limits is still considered acceptable and health risks are difficult to quantify. However, this limit is not fixed and could be influenced by the range of fish lengths, the area, and even the season of fishing. In order to further clarify the consumption of these wild fish, the consumption of wild fish of different lengths and collected from the waterways in urban and suburban areas were quantified successively [1,2]. However, without information on the seasonal variation in metal pollution in wild fish, it is difficult to determine the interseasonal fluctuations in the consumption of these wild fish. Given that the seasonal variations in metal accumulation in fish may significantly change the edible safety of fish [19,22], it is necessary to clarify the interseasonal fluctuations in the safe consumption of these wild fish, which is of great significance to determine the seasonal suitability for fish angling. As, Cr, Pb, and Hg are generally affected by anthropogenic emissions in nature, which may pose a threat to human health. Cu, Cd, Zn, and Se are relatively necessary to human beings, but are also affected by the environment, so they have received extensive attention. *Pseudohemiculter dispar* and *Cyprinus carpio* are most commonly caught and consumed by anglers in Liujiang River [2]. Therefore, the study aimed to investigate the seasonal variations in eight metals (As, Cr, Cu, Cd, Pb, Zn, Hg, and Se) in *Pseudohemiculter dispar* and *Cyprinus carpio* caught by anglers, and the evaluation of these angling fish could be useful to determine the more suitable and acceptable seasons for fish consumption. The study also aimed to use the current information as a means for environmental monitoring and to determine the effects of pollution.

### Highlight

- The mean concentrations of metals in fish are overall lower in winter.
- The effect of biological dilution should be more likely to exert its influence in winter.
- Metal bioaccumulation should be manipulated by living habitats in winter but impacted by feeding behavior in summer.
- The fish could be consumed freely in winter, but not entirely safely in summer.
- Winter should be treated as a suitable season for fish angling.

## 2. Materials and Methods

### 2.1. Study Sites and Fish Sampling

**Figure 1:**
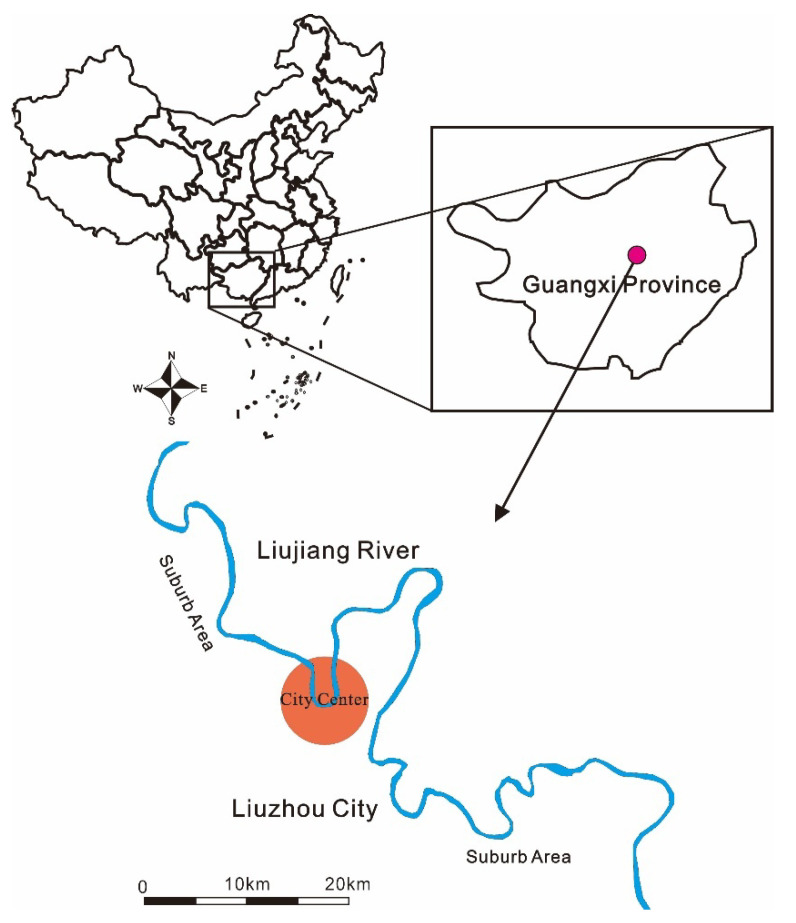
The study area in the city center of Liuzhou, China.

Liujiang River is the most important surface watershed in Liuzhou city with a total length of 272 km, which starts from Fengshan Town and flows through most of the functional areas of Liuzhou city, including industrial, commercial, and residential areas. Hence, the metal pollution of rivers distributed in Liuzhou city stems from industrial wastewater and domestic sewage. Based on previous studies, the city center is the most popular area for fish angling, where the gathering of many anglers is crowded along the river banks [1,2]. Therefore, the city center should be treated as a terrific area to conduct this study. Fish collection was conducted within a nearly 5 km radius in traditional commercial blocks along the banks of the river (109°33′ E~109°36′ E, 24°18′ N~24°21′ N) (red circle in Figure 1).

In order to assess the contamination and edible safety of angling fish, the fish samples were directly collected from anglers. *Pseudohemiculter dispar* and *Cyprinus carpio* are most commonly caught and consumed by anglers in Liujiang River [2], so they were incorporated into this study. *Pseudohemiculter dispar* and *Cyprinus carpio* are omnivorous, both of which prey on plankton, aquatic plants, and invertebrates. *Pseudohemiculter dispar* are pelagic fish, while *Cyprinus carpio* are benthic fish [24]. Although they live in different habitats, both of them like to live around the river banks. The low dynamic environment near the river banks causes aggregation of many nutrient substances in the sediment, which nourishes various plankton, aquatic plant, and invertebrate species, and then lures *Pseudohemiculter dispar* and *Cyprinus carpio* to feed around the river banks, especially in urban waterways [2]. That is also the reason why they are often hooked around the river banks. In winter, *Pseudohemiculter dispar* and *Cyprinus carpio* may hibernate in warm deep water to face the low temperature and degrade the eating rate to face the shortage of foods in cold weather [24], which also resulted in difficulties catching fish by anglers in extremely cold weather. However, the average winter temperature in Liuzhou is generally beyond 15 °C [25,26], which is relatively warm, so there is no absolute fish hibernation in deep waterways, but the transient inhabitation in deep waterways may occur on some days with the extremely low temperatures, confirmed by the fact that *Pseudohemiculter dispar* and *Cyprinus carpio* can still be caught by anglers in winter. Since the extremely low temperatures only appear in deep winter, we believe that the migration of *Pseudohemiculter dispar* and *Cyprinus carpio* from shallow waterways to deep waterways should only occur in deep winter when the temperature reaches the minimum. Once the temperatures rise again in winter, these fish will switch to feeding around the river banks again. The highest feeding rate of fish was mostly reported in early summer [19,22], which tightly related to their rapid growth with suitable temperature and abundant foods in early summer. Temperature affects the living and feeding behaviors of fish. Given the significant differences in living and feeding behaviors of fish in deep winter and early summer [19,20,22,27], which may cause a large variation in the bioaccumulation of metals in fish, the deep winter (15–20 February 2020) and the early summer (18–25 May 2019) were selected for the sample collection in this study. This design of sample collection will not only allow determination of the maximal seasonal variations in metal contamination among fish, but also help to clarify the regulation of living and feeding behaviors on metal bioaccumulation among fish. Given the more polluted fish primarily with lengths below 20 cm [1], this study only considered fish within the length of 20 cm. A total of 189 fish samples were collected in this study, among them, 76 and 113 fish samples were collected, respectively, in early summer and late winter. All fish samples were stored and transferred to the laboratory in a self-sealed polythene bag below −20 °C.

### 2.2. Sample Preparation and Analysis

The information about fish length and weight were recorded after sampling, while the species and autopsies of fish were identified in the laboratory (Table 1). The 50–100 g muscle tissues were extracted from the back of fish samples. The muscle tissues of more than three small samples with approximate lengths or weights were mixed to meet the minimal test weight requirement. Then, the muscle tissues were washed with Milli-Q water, freeze-dried for 72 h at −80 °C to obtain constant weight and powdered to uniform particle size with the grinder. ICP-MS and AFS-920 were respectively used to determine the concentrations of Cd, Cr, Cu, Pb, Zn, Se, Hg, and As [2]. The before and after freeze-dried weights of the fresh fish flesh were recorded to recover the metal contents in wet weight of fish. The descriptions of quality assurance and quality control were found in our previous literature [1].

### 2.3. Assessment Method

#### 2.3.1. Assessment of Metal Pollution

The metal pollution index (Pi) was applied to assess the contamination of metals among fish caught by anglers [1,2]. The Pi of Cd, Cr, Cu, Pb, Hg, and As were calculated using the following Equation (1):

Pi = Ci/Csi(1)

In this equation, Pi is the monomial pollution index of metal i; Ci and Csi are, respectively, the content and the threshold values of metal i in fish samples (mg/kg wet weight). The Csi of Pb, Cd, and Cr referred to the Maximum Residue Limits of Contaminants in Food of China [28], and the Csi of Cu, total Hg, and total As were in the light of the Safety Requirements for Non-environmental Pollution Aquatic Products of China [29]. 

The Pi values are divided into four pollution levels, namely Pi < 0.2, 0.2 < Pi < 0.6, 0.6 < Pi < 1, and Pi > 1, which, respectively, represent no significant pollution, minor pollution, moderate pollution, and severe pollution. The total metal pollution index (MPI) was used to assess the comprehensive metal pollution in fish. The concentration of metal n was represented by Cn, and MPI was calculated using the Equation (2):

MPI = (C1 × C2 × C3 × ……Cn)^1/n^ (2)

#### 2.3.2. Health Risk Assessment of Fish Consumption

The target hazard quotient (THQ) was applied to evaluate the health risks of those metals that exceeded the limits established by relative legislation [30]. THQ is the ratio between the exposure contaminant and the reference dose (RfD):

(3) $$\mathrm{THQ}=\frac{\mathrm{EF}\times \mathrm{ED}\times \mathrm{IRd}\times C}{\mathrm{RfD}\times \mathrm{BW}\times \mathrm{AT}}$$

The THQ value < 1 suggests that the exposed population is unlikely to suffer obvious adverse effects, while the THQ value >1 expresses that the level of exposure is beyond the Oral Reference Dose, so that effective interventions and protective measurements should be taken [31]. IRd, EF, ED, BD, AT, and RfD in Equation (3) are the ingestion rate of people (g/day), exposure frequency (days/year), exposure duration (year) and body weight (Kg), average time of exposure (day/year), and oral reference dose (RfD) (μg/kg/day), the values of which can be found in Miao et al. [2].

The calculation parameters of children and adults involved in Equation (3) are presented in Table 2. When calculating the THQ of Hg, the total concentration of Hg in the muscle tissue was assumed to be equal to that of MeHg, which has also been used by previous research [1,2]. The total target hazard quotient is equal to the sum of each metal THQ value, which can be presented as:

TTHQ = ∑THQ(4)

### 2.4. Statistical Analyses

SPSS 22 and Excel 2010 were used for the data analysis. The tables and figures were created using OriginPro 8 and Coreldraw X7.

## 3. Results and Discussion

### 3.1. The Concentrations of Metals in Fish

The concentrations of metals are given in Table 2. The mean concentrations of metals in fish are overall lower in winter than in summer, while the contents of Pb in fish is opposite, which is higher in winter and lower in summer. The decreased orders of metals in fish were not the same in different seasons, which were Zn > Cu > Se > Pb > Cr > As > Hg in winter and Zn > Cr > Cu > Se > Cd > As > Hg > Pb in summer, respectively. Although Zn was the most predominant metal in fish, regardless of season, the different orders of mean metals in fish still confirmed the varied bioaccumulation of metals between seasons. In winter, the metal concentrations in fish were significantly lower than their corresponding Maximum Residue Limit (MRL) [2], suggesting the relatively high safety of fish consumption in winter. In summer, although all mean concentrations of metals in fish were also lower than their corresponding MRL, the concentrations of Cr, Zn, and Cd in some fish were beyond MRL, which indicated the relatively low edible safety of fish in summer.

The bioaccumulation of metals in fish commonly follows the biological dilution effect [1], meaning that the increase in fat content can weaken or dilute the accumulation of metals among fish. Given the direct indicators of fish ontogeny, the weight and length of fish, have a direct correlation with their fat content [32], they have been widely used to uncover the biological dilution effect of metal accumulation in fish [1]. In this study, the correlation of metals was found to be significantly negative with the length and weight of fish in summer and winter, which demonstrated the vital roles of the biological dilution effect on the accumulation of metals in these angling fish, regardless of seasons. However, the correlations of most metals revealed that the effect of biological dilution should be more significant in winter than in summer. Given the prevalent fat consumption, instead of accumulation, in winter, it is a reason to believe that fat consumption may aggravate the effect of biological dilution on metal bioaccumulation in fish to some extent, which actually resulted from the shrinking of the enormous concentration gaps between fat and metals. However, it is worth noting that the weight loss would be revealed among fish with their fat consumption, which may inevitably increase the relative concentrations of metals at the same time. Given the affinity between the basal metabolic rate and fish weight [33], the higher consumption of fat among big fish may escalate their relative metal concentrations, on the contrary, the lower consumption of fat among small fish may impair the elevation of their relative metal concentrations in small fish. All of this suggests that the elevation of metal concentrations may also occur among big fish in winter, which definitely violated the effect of biological dilution. Therefore, there is reason to believe that fat consumption may also break down the effects of biological dilution in winter. However, the correlation of fish weight was only found to be positive with Cd in this study (Table 3), which suggested that the impacts of fat consumption on the biological dilution effect should be limited overall. Actually, the elevation of relative metal concentrations in fish that resulted from losing weight should be less related to their real contents, but heavily impacted by their residence time in fish. As a non-essential element, Cd could be difficult to involve in any metabolism functions, so the residence time of Cd was confirmed to be significantly longer than that of other metals [34,35]. The steady residence of Cd would inevitably minimize the influence of its metabolism, and then enhance its bioaccumulation in fish after fat consumption. Therefore, there is reason to believe that the bioaccumulation of metals among these angling fish should not entirely follow the rule of biological dilution in winter but could be also affected by their own metabolism of fat. Oppositely, fish commonly grow rapidly in summer so that their feeding and respiratory rates are high [36], both of which would exacerbate the intake of exogenous metals in fish [19], and then weaken the impacts of fat accumulation (or consumption) on metal bioaccumulation in fish. Therefore, the correlations between most metals, fish length, and weight were higher within the fish caught in summer. 

### 3.2. The Concentrations of Metals in Different Fish Species

**Figure 2:**
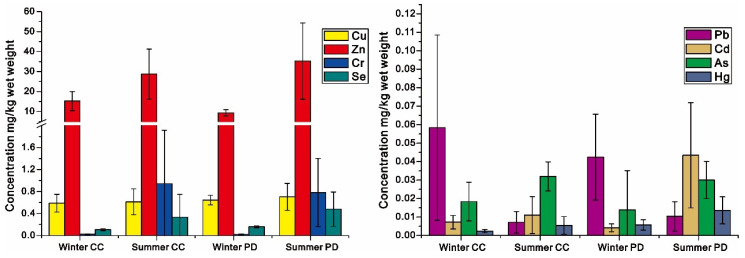
Concentrations of metals in *Cyprinus carpio* (CC) and *Pseudohemiculter dispar* (PD).

In winter, the concentrations of metals in *Cyprinus carpio* and *Pseudohemiculter dispar* decreased in the order (Figure 2) Zn > Cu > Se > Pb > Cr > As > Cd > Hg and Zn > Cu > Se > Pb > Cr > As > Hg > Cd, respectively, both of which are similar, but the small difference was only connected to the varied order of Cd and Hg, which suggested that the bioaccumulation of most metals between species was similar. However, in summer, the decreased orders of metals were Zn > Cr > Cu > Se > As > Cd > Pb > Hg for *Cyprinus carpio* and Zn > Cr > Cu > Se > Cd > As > Hg > Pb for *Pseudohemiculter dispar,* respectively. Though the variation in Zn, Cr, Cu, and Se is even between species, the big difference in As, Cd, Pb, and Hg suggested the bioaccumulation of metals between species was not even in summer.

The feeding behaviors should be treated as the most critical factor causing variation in the distribution of metals among fish in the same waterways [12]. The low feeding rate of fish in winter means that the structure of foods could have difficulty impacting the metal bioaccumulation in fish [21]. Therefore, the minor interspecific fluctuation in metal bioaccumulation in fish may highlight the dominant role of environmental stress on metal bioaccumulation in winter. With the impacts of the same environmental stresses, metal bioaccumulation in fish may be varied by their living habitats, which were well expressed by the overall higher metal concentration in *Cyprinus carpio* than that in *Pseudohemiculter dispar* in winter. *Cyprinus carpio* is benthic with a higher frequency of contact with sediments. Sediments were treated as the reservoirs of exogenous metals [37], and the exposure of contaminated sediments was bound to aggravate the metal bioaccumulation in *Cyprinus carpio*. However, *Pseudohemiculter dispar* is pelagic with a lower chance of contact with sediments, which also reduces the impacts of contaminated sediments on their metal bioaccumulation. Conversely, the feeding rate of fish is commonly high in summer [12], which would weaken the influence of environmental stress on the bioaccumulation of metals to some extent. Therefore, the metal accumulation in fish in summer could be not only determined by environmental stress, but also feeding behaviors. Although *Pseudohemiculter dispar* and *Cyprinus carpio* are omnivores, their feeding behaviors are not the same. With the influence of different feeding behaviors, the metal bioaccumulation in *Pseudohemiculter dispar* and *Cyprinus carpio* was found to be variable in summer. Different from winter, the food sources are abundant in summer so that feeding habits have a greater impact on metal bioaccumulation in fish than living habitats [12,22]. This was clearly expressed by the overall higher concentrations of metals in *Pseudohemiculter dispar* than that in *Cyprinus carpio*. *Pseudohemiculter dispar* is pelagic, generally living in the upper layer of the water, in particular, with a preference for foraging near river banks. In urban waterways with dense networks of sewage drainage, the lifestyle in shallow surface waters is bound to exacerbate the impacts of discharged sewage from river banks on the metal bioaccumulation in *Pseudohemiculter dispar*, due to the emission and diffusion of sewage starting from surface water. In contrast, *Cyprinus carpio* is benthic, primarily living in the lower layer of water. Although they may also hunt around the river banks, the time they stay in surface water must be less than *Pseudohemiculter dispar*, which decreases the influence of discharged sewage from river banks on their metal bioaccumulation to some extent, so that the metal concentration in *Cyprinus carpio* could be not compared with that of *Pseudohemiculter dispar*. Therefore, there is reason to believe that the metal bioaccumulation in fish should be more likely dominated by living habitats in winter, while the bioaccumulation should be more likely influenced by feeding behavior in summer.

### 3.3. The Degree of Metal Pollution in Fish

**Figure 3:**
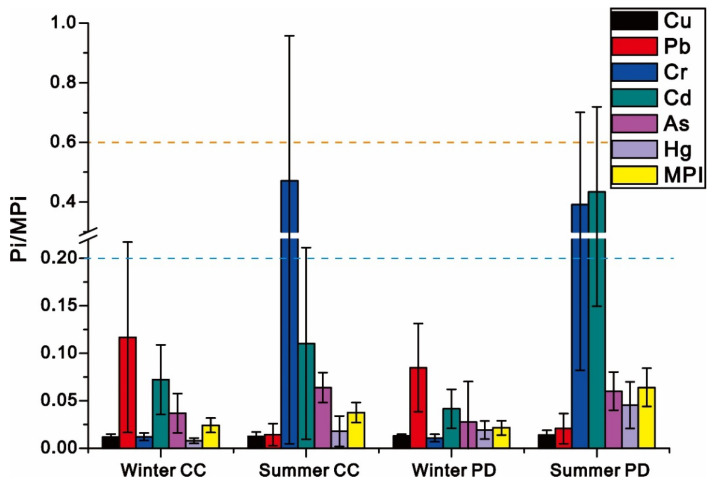
Pollution index (Pi) and multiple pollution indices (MPI) of metals in fish.

The metal pollution index was used for the assessment of metal pollution in fish and the corresponding results are shown in Figure 3. In winter, the decreasing orders of Pi values of metals in *Cyprinus carpio* and *Pseudohemiculter dispar* were as follows: Pb > Cd > As > Hg > Cu > Cr and Pb > Cd > As > Cr > Cu > Hg, respectively. Pb and Cd were the elements with the highest Pi values, among them, the high Pi values of Pb and Cd suggested that their contamination was relatively high in fish, corresponding to the high potential ecological risks of Pb and Cd in our previous studies [37,38]. The bioavailability of Pb and Cd in sediments of Liujiang River were reported to be significantly higher than other metals, both of which were beyond 50% [38]. Given the stay in warm deep water, fish had greater chances of exposure to sediments in winter and the frequent contact with sediments would conform the metal contamination of fish to that of sediments eventually, which also demonstrated the dominant roles of environmental stress on metal bioaccumulation in fish in winter. Although the contaminated sediments may aggravate the metal pollution in fish, the bioaccumulation of metals in fish could be not entirely determined by environmental stress, but also influenced by their feeding behavior. It is well known that the cold weather in winter degrades the feed and respiration rates of fish to a very low degree, which would substantially reduce the elevation in environmental stress on metal bioaccumulation in fish. It also explained that the contamination of Pb and Cd pollution in wild fish was not serious (Pi value below 0.2) despite the high contamination of Pb and Cd in sediments. Therefore, there is a reason to believe that, in winter, the effectiveness of environmental stress to enhance the metal pollution among wild fish should be limited. Similarly, although the living habitat can cause variation in the stress of metal pollution, in the context of the low feeding and respiration rates, the degree of metal pollution in fish would tend to be consistent, regardless of the fish species, confirmed by the approaching MPi values of *Pseudohemiculter dispar* and *Cyprinus carpio*. In general, the environmental stress in winter can only exert impacts on the types of metal pollution in fish but will struggle to significantly elevate the degree in fish. 

In summer, the Pi values of metals in *Cyprinus carpio* decreased in the order as follows: Cr > Cd > As > Hg > Pb > Cu (Figure 3). Cr has the highest pollution degree, while the Pi values of metals in *Pseudohemiculter dispar* decreased in the order as follows: Cd > Cr > As > Hg > Pb > Cu. Cd has the highest pollution degree. The average Pi values of Cr and Cd in these two fish species were significantly higher than 0.2, reaching 0.42 and 0.33, respectively. The corresponding maximum values of Pi were even beyond 1, which suggested that the fish were heavily contaminated with Cr and Cd in summer. The pollution status of metals in these fish generally reached moderate to severe pollution. Although the high Pi values of Cd suggested that these angling fish should be more likely to be impacted by the contamination of Cd regardless of the seasons, the overall higher Cd pollution in fish in summer, confirmed by the higher Pi and MPi of metals in summer, should be tightly related to the higher feeding rate in fish in summer. It is well-known that fish generally have higher respiration rates in summer. The higher respiration rate will directly influence the effectiveness of environmental stress on metal pollution in fish. Therefore, the metal pollution in fish was generally higher in summer than in winter, which was confirmed by the higher Pi and MPi of metals in summer. However, the bioaccumulation of metals in fish was not entirely determined by environmental stress; in particular, the feeding rate of fish was also higher in summer, which was expressed well by the widespread Cr contamination among wild fish in summer. Pb, instead of Cr, should be treated as a potential ecological risk in the watershed of Liujiang River, due to the large proportion of Pb in non-residual form [38]. Both of the dissolved and granular contents of Cr were low, especially the dominant proportion of Cr in the sediment was in the residual fraction of Cr (>80%) [38], which suggested that its bioavailability was extremely low. Fish could be difficult to contaminate with Cr under this background of low Cr. Therefore, there is reason to believe that metal pollution in fish should be also affected by their feeding behaviors. Both *Cyprinus carpio* and *Pseudohemiculter dispar* are small fish that prefer to live around the river banks and with environmental behavior of foraging around sewage outlets. Given the dense network of sewage drainage in urban waterways, the emission of eutrophic municipal sewage would significantly elevate the nutritional levels around the waterways with sewage discharge, which produces an aggregation effect in a variety of aquatic organisms, and then increases the frequency of small fish foraging around sewage emission outlets. Although municipal sewage is dominantly from domestic sewage, some industrial wastewater containing metals may be incorporated [39]. Therefore, the feeding behavior of foraging around sewage outlets was bound to exert impacts on metal bioaccumulation in fish. Cr is a very important and indispensable element for the production of steel, part manufacturing, and electroplating [40]. Automobile manufacturing and steel production are the main industries of Liuzhou [37]. Therefore, the wastewater of Liuzhou generally contains high concentrations of Cr [23]. The high feeding rate of fish in summer may increase the frequency of fish preying on aquatic biota around wastewater outlets, the accumulation of Cr in wild fish of Liujiang River could have inevitably increased, and then elevated the contamination of metals in fish. However, the significant differences in MPi values between *Pseudohemiculter dispar* and *Cyprinus carpio* indicated that the influence of living habitats on the metal pollution in fish should not be ignored. Given the fact that the diffusion of discharged sewage is from the surface layer of water to the bottom layer of water, the pelagic fish should suffer from more direct stress of sewage than the benthic fish, so that the contamination degree should be higher in pelagic fish, which corresponds to the higher MPi of *Pseudohemiculter dispar*. Therefore, there is reason to believe that both living habitats and feeding behaviors of fish could, together, impact the metal contamination in summer.

In general, the Pi values of most heavy metals were high in summer and low in winter. For Pb, although the Pi value in winter was higher than that in summer, MPi of summer was still found to be 2.5 times higher than that of winter, indicating that the elevation of some metal pollution in winter may mean that it is difficult to reverse the overall low metal pollution in wild fish. 

### 3.4. The Assessment of Potential Health Risks Associated to Metals in Fish

**Figure 4:**
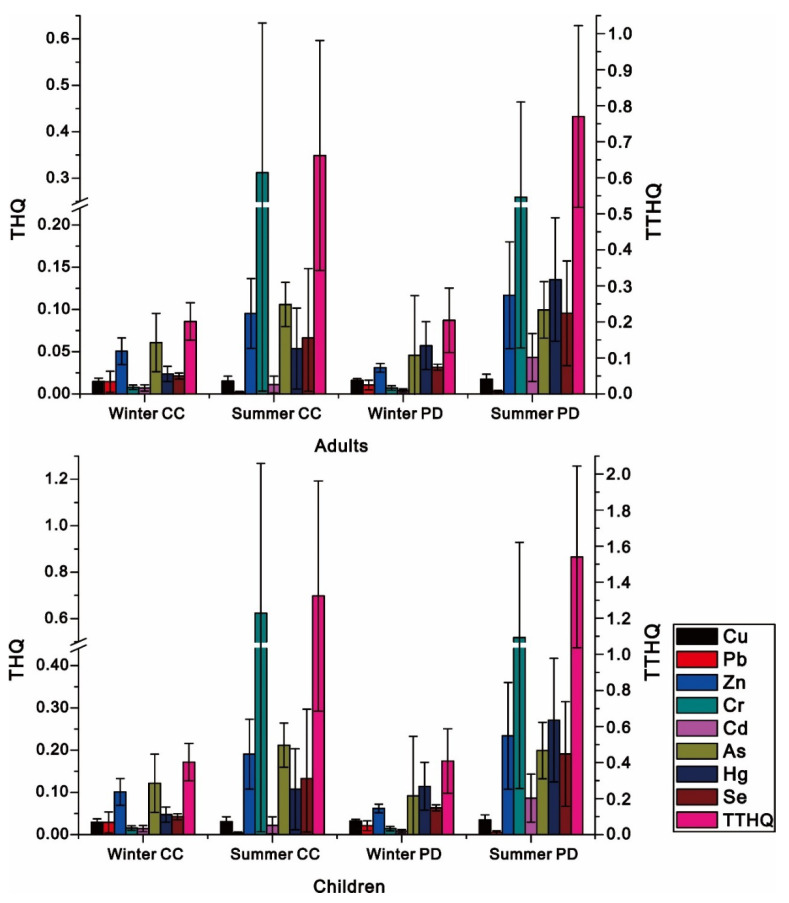
Target hazard quotients (THQ) of metals in fish.

The potential health risks of fish consumption were calculated in accordance with adults and children, as shown in Figure 4. In winter, THQ values of metals in *Cyprinus carpio* and *Pseudohemiculter dispar* decreased in the orders As > Zn > Hg > Se > Cu > Pb > Cr > Cd, Hg > As > Se > Zn > Cu > Pb > Cr > Cd, respectively. The maximal THQ values of *Cyprinus carpio* and *Pseudohemiculter dispar* were found to be As and Hg, respectively, indicating that the health risk of consuming these fish should be more related to As and Hg. However, it is worth noting that the contamination of As and Hg was found to be lower in this study, which suggested the health risk of metals should be not directly connected to their contamination. Actually, the health risks of metals are tightly related to their corresponding toxicity and tolerance in humans [41]. As and Hg are highly toxic, which could be tolerated only in extremely low levels [42,43], on the contrary, although Pb and Cd are also toxic, the levels that human can tolerate are much higher than that of As and Hg [1]. It is well explained that the higher contamination of Pb and Cd in fish fails to exert any impacts on elevating their corresponding THQ. The TTHQ value of *Pseudohemiculter dispar* was close to that of *Cyprinus carpio*. Although the TTHQ value of children was much higher than that of adults, both of their TTHQ values were significantly lower than 1, indicating that the consumption of fish in winter was generally safe and may not pose a threat to public health. In summer, the THQ values of metals in *Cyprinus carpio* and *Pseudohemiculter dispar* decreased as follows: Cr > As > Zn > Se > Hg > Cu > Cd > Pb and Cr > Hg > Zn > As > Se > Cd > Cu > Pb, respectively. The THQ value of Cr was found to be the highest in these two fish species. Although the mean THQ values of metals were all below 1, cases where the THQ values were greater than 1 could be also found in this study, which only resulted from the higher THQ of Cr, especially for children. The maximum THQ value of Cr even exceeded 1.5, indicating that there was enormous risk underlying the consumption of some fish. Both the THQ values and Pi values of Cr are higher than other metals, regardless of fish species, which demonstrated the strong impact of metal contamination in fish on their consumption safety. For TTHQ values, the TTHQ values of *Pseudohemiculter dispar* and *Cyprinus carpio* were very close in winter, which suggested the health risk of fish consumption could be hard to decrease by only relying on species changes in winter. In summer, the TTHQ value of *Pseudohemiculter dispar* was higher than that of *Cyprinus carpio*, indicating a higher health risk of fish consumption. In general, the TTHQ value of wild fish was significantly lower than 1 in winter, indicating a low risk of fish consumption. Therefore, the consumption of wild fish should be safe in winter. However, the TTHQ value of wild fish was relatively high in summer. For both adults and children, the TTHQ values of some wild fish were found to be higher than 1 in summer, which demonstrated that fish consumption was of great health risk in summer. For adults, the health risks of fish consumption should be relatively low due to the high tolerance to metal pollution, which corresponds to the low TTHQ of adults. In contrast, the tolerance of children to metals was considered to be low due to their light body weights [44]. Therefore, the consumption of wild fish is commonly associated with higher health risks for children. The maximum values of TTHQ of some wild fish even reached 3.4 in summer, the average value of which was also significantly beyond 1, indicating that the consumption of wild fish would be more likely pose a great threat for the health of children. Therefore, it is better to avoid the consumption of wild fish by children in summer.

**Figure 5:**
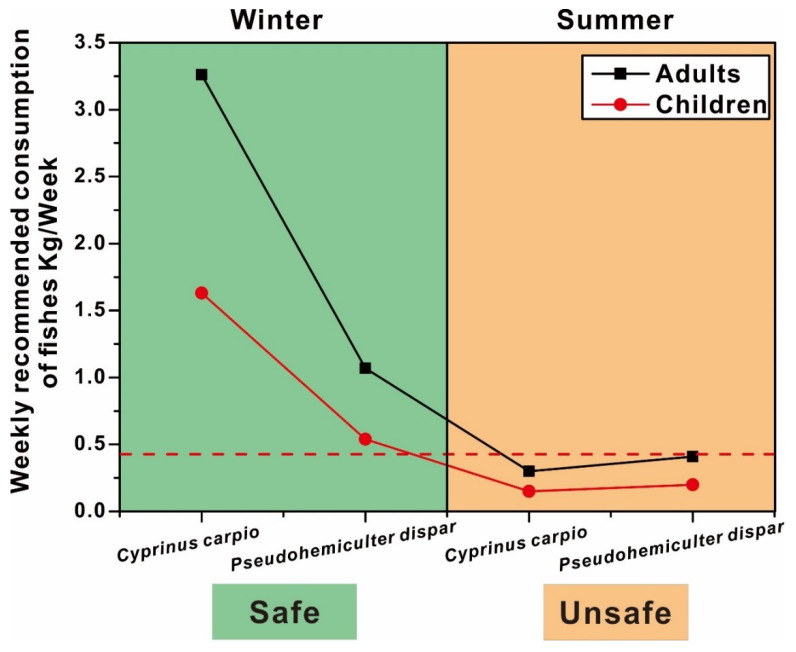
Weekly recommended consumption of fish from different sample sites, kg/week.

The amount of fish consumption among urban residents in Guangxi, China, was reported to be 0.42 kg/week in a previous study [45]. However, eating angling fish in accordance with this standard would expose adults and children to various degrees of health risks. In order to further quantify the fish consumption, the weekly maximum allowance of fish consumption was calculated following our previous studies [2]. Consequently, the contamination of As and Cr were, respectively, determined to be the greatest drawback of fish consumption in winter and summer, just as their THQ contributed most to TTHQ. For winter, the safe consumption of *Cyprinus carpio* should be a maximum of 3.26 kg/week for adults and 1.63 kg/week for children, respectively, while the safe consumption of *Pseudohemiculter dispar* should be no more than 1.07 kg/week for adults and 0.54 kg/week for children (Figure 5). Although the fish consumption in children was significantly lower than adults, they are all far beyond 0.42 kg/week, which suggested that these angling fish caught in winter could be considered to be safe for consumption by anglers. However, in summer, the edible safety of angling fish was not as higher as that in winter, which suggested the overall lower permissible consumption amount of angling fish in summer. The suitable consumption amounts of *Cyprinus carpio* would be below 0.3 kg/week for adults and 0.15 kg/week for children, respectively, while the safe consumption amounts of *Pseudohemiculter dispar* should not exceed 0.41 kg/week for adults and 0.20 kg/week for children, respectively. They are all significantly below 0.42 kg/week, which suggested that these angling fish caught in summer were less safe to eat and should be avoided, particularly for children. In general, the consumption amounts of fish caught in winter were 2.61 times higher for *Pseudohemiculter dispar* and 10.87 times higher for *Cyprinus carpio* than that caught in summer. Therefore, winter should be treated as a suitable season for fish angling.

## 4. Conclusions

The mean concentrations of metals in fish are overall lower in winter. Only Cr, Zn, and Cd in some fish caught in summer were beyond the MRL. The biological dilution effect exerted its influence on metal bioaccumulation in fish in winter. The similar distributions of metals in winter suggested that metal bioaccumulation was influenced by living habitats, while the inconsistent distribution of metals in summer suggested metal bioaccumulation was impacted by feeding behavior. The Pi values of metals were below 0.2 in winter, suggesting no metal contamination in fish, but most fish were found to be generally contaminated by Cr and Cd in summer, confirmed by their Pi > 0.2. The fish caught in winter could be consumed safely in accordance with TTHQ < 1, while the consumption of fish caught in summer was considered to be not entirely safe, particularly for children, mainly due to TTHQ values generally beyond 1. Given the higher weekly recommended consumption of fish in winter, winter should be treated as a suitable season for fish angling.

## Figures and Tables

**Table 1:** Detailed information about the collected fish.

Season	Species	Num	Length	Weight	Feeding Habit	Habitat
			cm	g		
Winter	*Cyprinus carpio*	51	8.2–19.7	8.1–127.1	Omnivore	Demersal
	*Pseudohemiculter dispar*	62	9.3–19.9	5.3–88.7	Omnivore	Pelagic
Summer	*Cyprinus carpio*	32	8.1–19.5	8.9–135.2	Omnivore	Demersal
	*Pseudohemiculter dispar*	44	8.1–19.7	5.1–96.3	Omnivore	Pelagic

**Table 2:** The concentrations of metals in fish collected from anglers.

Season		Cu	Pb	Zn	Cr	Cd	As	Hg	Se
		mg/kg							
Winter		0.388–1.007	0.013–0.228	6.942–25.448	0.01–0.047	0.001–0.016	0.002–0.117	0.001–0.02	0.071–0.217
		0.627	0.048	11.274	0.022	0.005	0.015	0.005	0.142
Summer		0.249–1.38	0.001–0.044	8.532–77.213	0.04–4.226	0.002–0.113	0.001–0.056	0.002–0.037	0.052–1.971
		0.673	0.009	33.103	0.836	0.033	0.031	0.011	0.432
Maximum Residue Limits (MRL)	China	50	0.5	-	2	0.1	0.5	0.3	-
	International	30	0.5	50	8	0.05	1	0.5	2

**Table 3:** The correlations between length, weight, and metal concentrations.

	Weight	Cu	Pb	Zn	Cr	Cd	As	Hg	Se
	Winter								
Length	0.598 **	−0.129	−0.217	−0.573 **	−0.302 **	0.045	0.133	0.192	0.252 *
Weight	-	−0.314 **	−0.031	0.018	−0.145	0.308 **	0.142	−0.275 *	−0.419 **
	Summer								
Length	0.895 **	−0.349 *	−0.075	−0.189	−0.247	−0.224	−0.028	−0.055	0.065
Weight	-	−0.368 **	−0.085	−0.212	−0.244	−0.284 *	−0.071	−0.207	−0.046

** Correlation is significant at 0.01 level (2-tailed); * correlation is significant at 0.05 level (1-tailed).

## Data Availability

The original contributions presented in the study are included in the article, further inquiries can be directed to the corresponding author.
